# Comparison of Interfaces Between In Situ Laser Beam Deposition Forming and Electron Beam Welding for Thick-Walled Titanium Alloy Structures

**DOI:** 10.3390/mi15111383

**Published:** 2024-11-15

**Authors:** Pingchuan Yang, Fei Li, Zongtao Zhu, Hui Chen

**Affiliations:** 1 Chengdu Aircraft Industrial (Group) Co., Ltd., Chengdu 610091, China; lixinsrkj@163.com (P.Y.); leoscf132@163.com (F.L.); 2 Key Laboratory of Advanced Technologies of Materials, Southwest Jiaotong University, Ministry of Education, Chengdu 610031, China; 3 High-End Equipment Advanced Materials and Manufacturing Technology Laboratory, Chengdu 610031, China

**Keywords:** electron beam welding, laser additive manufacturing, titanium, microstructure, texture

## Abstract

An investigation was conducted on electron beam-welded and additively manufactured joints on a thick-walled titanium alloy utilizing in situ laser beam deposition and electron beam welding techniques. The surface morphology, microstructural characteristics, and mechanical properties of both joint types were comprehensively analyzed using stereomicroscopy, scanning electron microscopy (SEM), microhardness and tensile strength testing, and electron backscatter diffraction (EBSD) techniques. The electron-beam-welded joint exhibited distinct fusion and heat-affected zones, whereas the laser-beam-deposited joint exhibited a smoother surface that was free from excess spatter. Both joints featured a sharp microstructural boundary with a pronounced hardness gradient across the interface, lacking a gradual transition area. During tensile testing, both joint types demonstrated a mixed brittle-ductile fracture mode; however, the electron beam-welded joints surpassed the laser-beam-deposited joints in terms of tensile strength, achieving over 1183 MPa with an elongation of more than 7.3%, compared to 1123 MPa and 5.9% elongation, respectively.

## 1. Introduction

Titanium and its alloys, which were developed as crucial structural metals with high specific strength, corrosion resistance, biocompatibility, and comprehensive mechanical properties in the 1950s, have been widely used in various fields, such as aerospace, marine engineering, and medical device industries [1,2,3]. As the aerospace industry places greater significance on utilizing titanium structure components like turbine engine blades, compressor rotors, and flow control valve discs [4,5], stricter demands have emerged for the strength and precision of titanium alloy parts. In practical engineering applications, titanium alloys are susceptible to considerable wear and damage. Due to the high cost of titanium alloys, direct replacement of parts is often economically impractical, making repair methods preferable. Conventional repair methods typically involve welding, followed by precision grinding, to restore the component’s original shape. A novel approach involving additive manufacturing [6,7,8,9] presents a more cost-effective and efficient method for directly repairing components, thus eliminating the need for part replacement.

Additive manufacturing technology has been extensively applied in the fabrication of titanium, aluminum, iron-based alloys, and other metallic components owing to its rapid production, cost-effectiveness, high flexibility, and seamless integration capabilities [10]. Notably, titanium castings with a remarkable 99.5% densification can be achieved through the hot isostatic pressing (HIP) process, surpassing the properties of conventional castings [11]. Furthermore, prior research has validated that intricate titanium components produced via laser additive manufacturing (LAM) exhibit mechanical properties comparable to those of forgings [12]. Consequently, the advancement and widespread adoption of metal additive manufacturing technology have ushered in innovative production processes for titanium castings, while also justifying the practical exploration of large-scale and high-volume additive manufacturing applications on the surfaces of titanium structural components.

Capitalizing on the merits of additive manufacturing, academic endeavors have focused on composite manufacturing techniques that integrate titanium alloy additive manufacturing with traditional forging technology. O. Dolev [13] discovered that the hybrid TC4 structure, achieved by selective laser melting (SLM) on pre-forged TC4, exhibited superior fracture toughness compared to either the purely additive-manufactured or forged alloy. Additionally, Du [14] utilized cold metal transfer additive manufacturing technology to fabricate TC4-DT specimens, revealing that the overlap zone of TC4-DT attained the highest yield strength and lowest work hardening coefficient, attributable to the influence of β grain boundaries and the <0001>α//X orientation texture.

Nevertheless, the additive manufacturing mentioned above can be considered a form of dissimilar metal joining to some extent, as it involves connecting the same material in different states. Welding is one of the most effective methods for connecting dissimilar materials. Compared with other welding methods, electron beam welding (EBW) is considered the preferred welding technology for thick titanium plates due to its higher heat source energy density, larger depth-to-width ratio of the weld, and relatively less heat-affected zone [15,16,17]. Hence, it is of certain research significance to compare titanium alloy interfaces deposited by laser additive manufacturing with welded interfaces by electron beam welding. In this study, butt EBW was carried out between 47 mm forged Ti-6Al-4V (TC4) plates and an additive-manufactured TC4 plate with the same thickness, and laser additive manufacturing was performed directly in the thickness direction of another forged titanium plate to obtain a molded component joint that matched the EB-welded joint size. The microstructures of the welds and interfaces of the joints were studied by metallurgical microscopy and electron backscatter diffraction (EBSD). The tensile strength and microhardness distribution at the interface of the two joints were investigated, which laid the foundation for the further possibility of additive manufacturing and EBW technology to obtain the optimal structural design of titanium components with functional priority.

## 2. Experimental Materials and Methods

**Figure 1:**
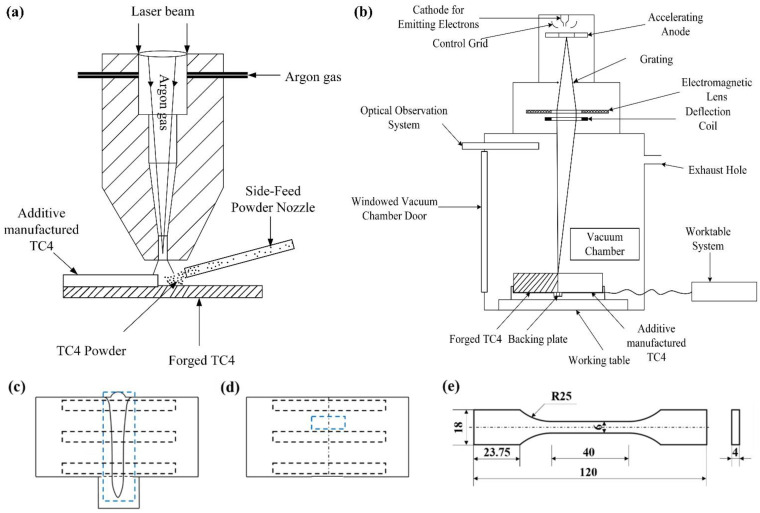
Schematic diagrams illustrating (**a**) the EBW of thick titanium plates with a backing plate, (**b**) additive manufacturing on a forged TC4 plate, (**c**,**d**) the location of the regions for metallographic examination and strength testing (viewed from the two cross-sections), and (**e**) the dimensions of the tensile samples (unit: mm).

Two different connection joints were investigated in this study. The original deposited TC4 titanium alloy obtained by laser deposition was cut into plates with dimensions of 200 mm × 65 mm × 47 mm by wire cutting, which was used for vacuum electron beam welding along the length direction with forged TC4 titanium alloy plates of the same size. Laser deposition was performed on the side of another forged TC4 plate, i.e., the plane where the length and thickness of the plate were located, to obtain an additive-manufactured titanium joint. Before EBW or additive manufacturing, the joining surfaces of the plates were sanded to remove oils and oxides, and cleaned with acetone. Figure 1a,b show schematic diagrams of EBW and 3D printing, respectively, and the corresponding experimental parameters are described in detail in Table 1. In this study, the joint thickness direction was designated as ND, the joint length direction (parallel to the welding direction) was denoted as TD, and the joint width direction (perpendicular to the welding direction) was referred to as RD. Similarly, to ensure consistency in subsequent experimental and analytical testing, the additive-manufactured joints, TD, ND, and RD were defined as parallel to the scanning direction, thickness direction of the additive manufacturing joint, and deposition direction, respectively.

**Figure 2:**
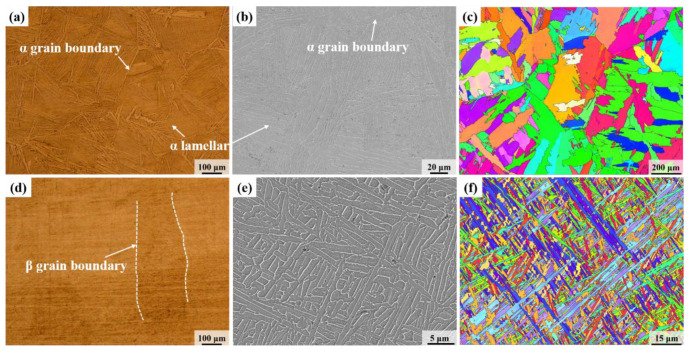
Microscopic images of base metals: (**a**–**c**) microstructure revealed by OM, SEM image, and IPF coloring map of forged TC4, (**d**–**f**) microstructure revealed by OM, SEM image, and IPF coloring map of additive-manufactured TC4.

Due to the span of the EBW joints in the thickness direction, microstructure observations and tensile strength tests were performed on different parts of the joint, i.e., the upper, middle, and bottom parts. For the additive-manufactured joint, the metallographic sample was cut in the middle of the connection interface, while the tensile samples were cut on both sides and the middle along the interface. The sampling positions and sizes of the metallographic and tensile specimens are illustrated in Figure 1c–e. The mechanically polished metallographic samples were etched for 10–15 s using an etching solution with a ratio of 2 mL HF. 6 mL HNO_3_ and 92 mL H_2_O, followed by observation of the microstructure under a Zeiss-AIM microscope. As shown in Figure 2, the microstructure of the forged TC4 base metal is primarily characterized by grain boundary α (α_GB_), lamellar α phase, and intergranular β phase, while the microstructure of the additive-manufactured TC4 consists of coarse β columnar grains and intragranular needle-like α′ martensite. Under low magnification (as shown in Figure 2d), clear β grain boundaries were observed, which can be attributed to the fact that the temperature during laser additive manufacturing exceeds the α → β phase transition temperature of the TC4 titanium alloy. As a result, the α phase transforms into the β phase, and a significant amount of fine needle-like martensite forms within the β grains due to the relatively rapid cooling rate. Under high magnification of a scanning electron microscope (SEM), the microstructure of additive-manufactured TC4 consisted of basket-weave structures, with α phase growth in different directions and intersecting each other [18], as shown in Figure 2e. The EBSD data of the two joints were processed using the HKL Channel 5 software to analyze the evolution of the microstructure and texture in the two connection modes. Tensile strength tests were performed on a WDW3100 (Guangzhou Guangjing Precision Instruments Co., Ltd., Guangzhou, China) electronic tensile machine at a rate of 4 mm/min, and the fracture morphology was observed under a Gemini 300 scanning electron microscope. Then, a microhardness test was conducted to reveal the microhardness distribution in various regions of the two joints.

## 3. Results and Discussion

### 3.1. Metallographic Characterization

**Figure 3:**
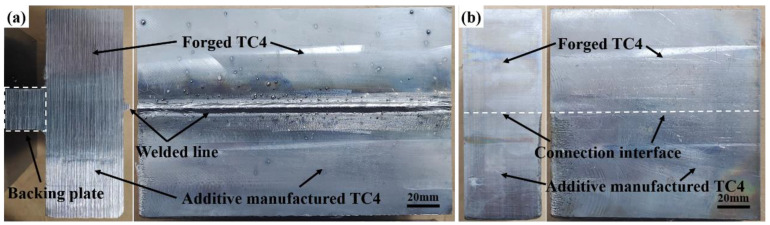
The macrostructure of (**a**) the EB-welded joint and (**b**) the additive-manufactured joint.

The macroscopic and microscopic morphology evaluations were conducted on the cross-sections of the EB-welded joint and additive-manufactured joint. Figure 3 illustrates the macroscopic morphologies of the two joints. As shown in Figure 3a, the surface of the EB-welded joint exhibited a small amount of spattering and a slight weld undercut was observed at the end of the joint. In addition, no welding defects such as porosity or cracks were detected on the surface. The additive-manufactured joint shown in Figure 3b exhibits no visible defects, with a smooth surface and a clear interface.

**Figure 4:**
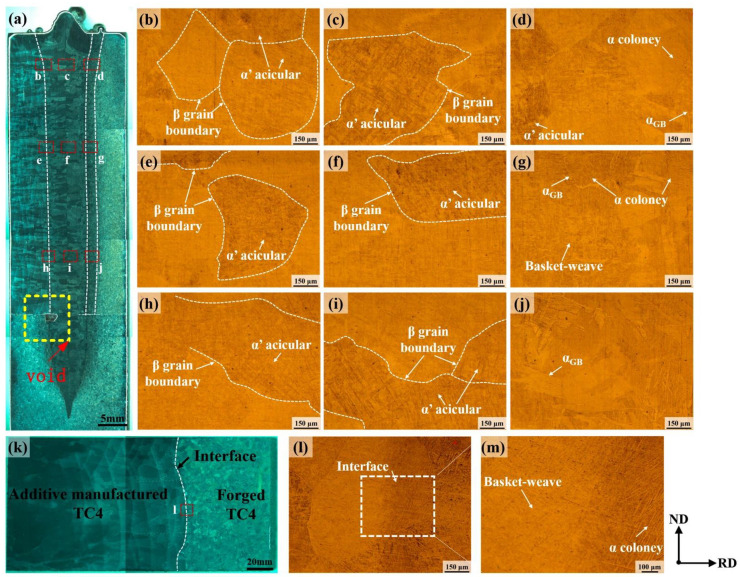
OM images of two joints: an overview of (**a**) the EB-welded joint and (**k**) the additive-manufactured joint; (**b**–**d**), (**e**–**g**), and (**h**–**j**) represent the top, middle, and bottom of the EB-welded joint, respectively; and (**l**,**m**) represents the interface.

Figure 4 presents the microstructure of the cross-section of the two joints under low magnification using an optical microscope. Due to the larger sample size of the electron-beam-welded joint, the image was captured and stitched multiple times, as shown in Figure 4a. Similarly, a cross-sectional image of the additive-manufactured joint, as shown in Figure 4k, was obtained through two courses of image collection. No defects were observed in the weld zone in Figure 4a; however, a larger void was observed on the backing plate side. The formation of the void was speculated to be related to the formation mechanism of the electron beam weld, where vaporized metal or other gases at the root of the weld experienced resistance from the liquid metal expelled by the electron beam during the welding process, and residual gases were trapped at the bottom of the weld during rapid cooling. Owing to the void being positioned on the backing plate side, its presence did not exert any discernible effect on either the microstructural features or performance characteristics of the weld zone. The EB-welded joint exhibited a relatively small overall width variation above the backing plate, and the relatively wider size of the upper part of the weld resulted in a “T”-shaped nail-like feature of the joint. The occurrence of tip defects was observed in the substrate region, indicating that the welding parameters employed in the experiment effectively achieved complete penetration welding of 47 mm thick TC4. Based on the basic microstructure characteristics within different regions of the joint, the EB-welded joint was roughly divided from left to right into the following areas: 3D-printed titanium alloy base metal, heat-affected zone of 3D-printed titanium alloy side (3D-HAZ), fusion zone (fusion zone), heat-affected zone of forged titanium alloy side (Forged-HAZ), and forged titanium alloy base metal. As shown in Figure 4a, the fusion zone was located at the center of the weld and was characterized by coarse elongated columnar crystals. The columnar crystals on both sides of the fusion zone center gradually increased in angle from top to bottom, eventually exhibiting a distribution perpendicular to the weld thickness direction. Moreover, the grain size in the top region of the fusion zone was larger than those in the middle and bottom regions. This could be attributed to the higher intensity of electron beam irradiation in the top region of the fusion zone, whereas the middle and bottom regions exhibited relatively less heat absorption and faster cooling rates. Consequently, solidification of the liquid metal in the weld pool commenced from the bottom and progressed upward. Enhanced energy absorption occurred in the top region of the weld pool under the high-temperature influence of the electron beam, resulting in a more pronounced coarsening phenomenon during solidification. According to the characteristics of grain structure, size, and location, the Forged-HAZ is delineated in Figure 4a, while the 3D-HAZ could not be distinctly differentiated as its microstructure closely resembled that of the fusion zone, it was difficult to label its location, as shown in Figure 4a. Hence, further microscopic analysis was required for assessment.

Figure 4b–j shows the microstructures of different regions of the EB-welded joint. Figure 4c,f,i show the center regions of the fusion zones at the top, middle, and bottom parts of the weld, respectively. Upon comparison, it was found that the microstructure characteristics of the fusion zones in these three regions were quite similar. The early precipitated Ti-β phase boundaries could be distinctly identified in these three micrographs of the fusion zones, with the β phase transforming into acicular α′ martensite within the boundaries. This was attributed to the concentrated energy impact of the electron beam during the welding process, which caused the β phase in the fusion zone to undergo a phase transformation at a high temperature and a rapid cooling rate, resulting in the formation of fine acicular α′ martensite [19,20]. From the comparative analysis of Figure 4b,e,h, there is no difference between the microstructure on the 3D-HAZ side and that of the fusion zone. Compared to the fusion zone, the β phase grain size was slightly smaller in the 3D-HAZ, with a short and dispersed distribution of acicular α′ martensite. This was presumed to be caused by the relatively lower heating temperature in the 3D-HAZ compared with that in the fusion zone. Figure 4d,g,j shows the top, middle, and bottom regions of the Forged-HAZ of the joint, respectively. The microstructure showed no variation along the joint thickness direction; however, there was a transition parallel to the joint thickness direction. As illustrated in Figure 4d, the microstructure adjacent to the fusion zone on the left side of the figure was mainly composed of acicular martensite, and as it moved away from the weld center, both the content and size of the martensite decreased. In addition, a grain boundary α phase (α_GB_) was formed, with lamellar α colonies distributed on both sides of the α_GB_ phase. This was a result of the formation of a discontinuous α_GB_ phase at the β grain boundaries during the cooling process of the liquid metal when the temperature decreased to the α-β phase region. Subsequently, the α phase nucleated at the interfaces between the grain boundaries of β and α_GB_ and then extended into the β phase in a consistent direction, leading to the formation of α colonies [21,22].

Figure 4k shows the macroscopic morphology of the additive-manufactured joint. A clearly defined interface appeared between the two base metals without the presence of any defects such as pores or inclusions. The difference in the microstructure between the two sides of the interface can be observed in Figure 4l. Upon magnification of the interface (Figure 4m), it was shown that on the left side interface, the microstructure consisted of acicular martensite morphology, while the size of the martensite structure decreased, and α colonies appeared gradually near the interface. On the right side of the interface, the microstructure transitioned to the lamellar α-phase and eventually approached that of the forged titanium alloy base metal.

### 3.2. Microhardness in Different Regions of the Joint

**Figure 5:**
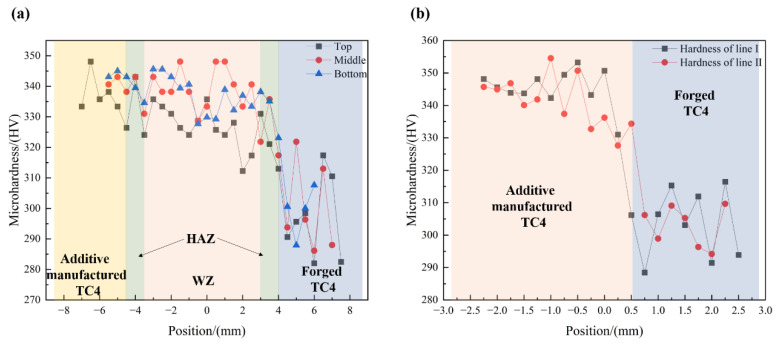
The microhardness of the (**a**) EB-welded joint and (**b**) additive-manufactured joint.

During microhardness testing, the spacing between test points was set at 0.5 mm for the EB-welded joint due to its larger size. For the additive-manufactured joint without a distinct transition region, a small spacing of 0.25 mm was chosen to ensure accuracy. Each region was tested with two rows of hardness test points, and the average value of the test results of the two rows was taken as the microhardness distribution for that region. Figure 5a presents the average microhardness results of the top, middle, and bottom regions of the EB-welded joints, while Figure 5b shows the hardness test results of two rows of the additive-manufactured joint. It was found that the microhardness of additive-manufactured titanium alloy base metal was generally higher than that of the forged titanium alloy and slightly higher than that of the fusion zone. This is attributed to the formation of numerous fine needle-like martensitic structures in the TC4 alloy obtained through additive manufacturing under rapid solidification conditions [23]. For the additive-manufactured joint, no significant microhardness gradient or thermally affected zone was observed on either side of the joint interface. Based on the microstructure shown in Figure 4, it was preliminarily inferred that the additive manufacturing process under the current conditions did not lead to the formation of heat-affected zones with significant microstructural differences.

### 3.3. Tensile Strength and Fracture Morphology

**Figure 6:**
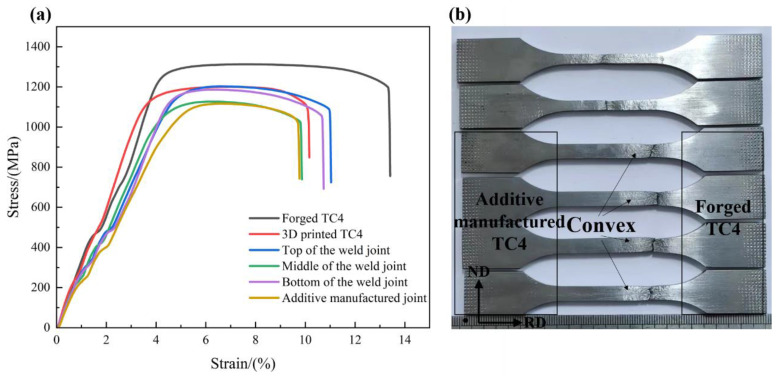
(**a**) The true stress-strain curve and (**b**) the macroscopic morphology of the fractured samples after the tensile test, from top to bottom: forged TC4, additive-manufactured TC4, top, middle, and bottom of the weld joint, respectively, and the additive-manufactured joint.

Figure 6 illustrates the stress-strain curves of the different regions of the two joints. For comparison, the tensile properties of the two base materials were also tested. Table 2 presents the obtained tensile strength and elongation values, which represent the average results of repeated experiments. The tensile strength was ranked in descending order as follows: forged TC4 (1314 MPa) and additive-manufactured TC4 (1242 MPa). The top of the electron-beam-welded joints (1211 MPa), bottom of the electron-beam-welded joints (1183 MPa), middle of the electron-beam-welded joints (1165 MPa), and additive-manufactured joints (1123 MPa). The tensile strength and elongation of the additive-manufactured joints were relatively lower than those of the electron-beam-welded joints, measuring 5.94%. It can be seen that the tensile strength of the additive manufacturing joints was slightly lower than that of the electron beam welding joints. Additionally, the elongation of the additive manufacturing joint was only 5.9% compared to the 7.8% elongation of the electron beam welding joint.

**Figure 7:**
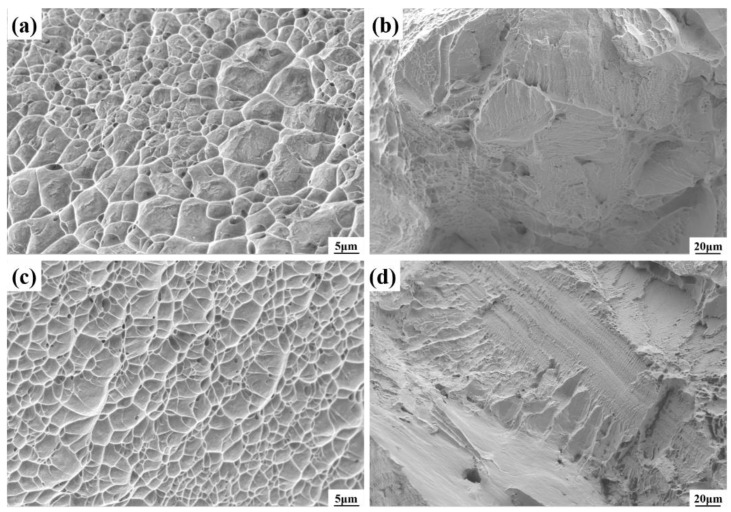
The Scanning electron microscope fracture morphology of (**a**,**b**) the EB-welded joint and (**c**,**d**) the additive-manufactured joint.

Figure 6b shows the corresponding tensile samples after the tensile test; it is the event that failure of both joints occurred near the forged titanium alloy region, with a significant necking phenomenon observed along the gauge section adjacent to the fracture location. The fracture morphology in Figure 7 indicates that the fracture formation of both the electron-beam-welded and additive-manufactured joints exhibited a hybrid fracture pattern of ductile and brittle fracture modes. Upon comparing Figure 7a,c at an equivalent SEM magnification, the tensile specimens of the EB-welded joint displayed more pronounced and deeper dimples, indicating the superior toughness of the joint, which is consistent with the experimental results shown in Table 2.

### 3.4. The Effect of Interface Microstructure on Joint Strength

**Figure 8:**
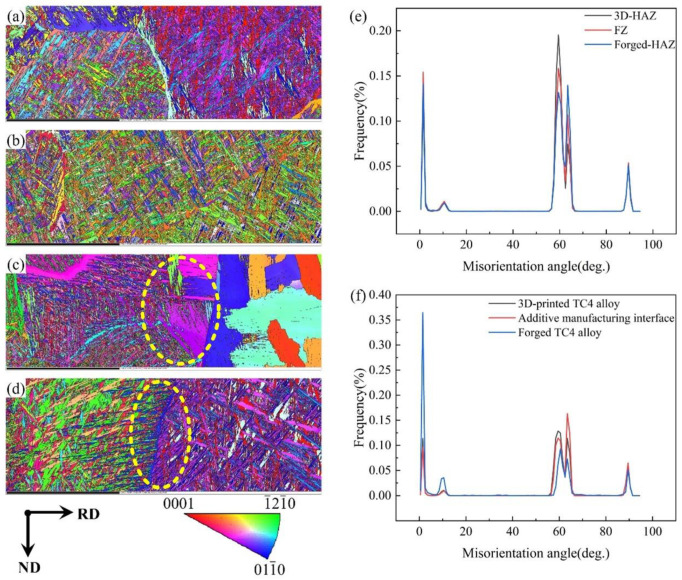
(**a**–**c**) IPF maps of the 3D-HAZ, FZ, and Forged-HAZ of the top region of the EB-welded joint, respectively; (**d**) IPF map for the interface of the additive-manufactured joint; and (**e**,**f**) orientation distribution map for the two joints.

As is shown in Figure 8a–c are the IPF maps of 3D-HAZ, FZ, and Forged-HAZ of the top region of the EB-welded joint, respectively; Figure 8d is the IPF map for the interface of the additive-manufactured joint, and Figure 8e,f are the orientation distribution maps for the two joints. A comparison of Figure 8c,d reveals significant differences in the grain structures on both sides of the electron beam weld joint. The left side of the joint consists of fine needle-like structures interwoven into a network, while the right side, influenced by the welding thermal cycle, is composed of large equiaxed grains. No distinct interface exists between these two structures; instead, the needle-like structure penetrates into the equiaxed grains, forming a pinning effect that enhances the joint’s strength. In the joint produced by 3D printing, both sides consist of fine needle-like structures with different grain orientations. As shown in Figure 8f, the left side of the interface contains a higher proportion of small-angle grains, while the right side displays a significantly reduced proportion of such grains. Near the interface, a small number of needle-like grains extend into the forged TC4 titanium alloy. The structures on both sides adhere at the interface, which accounts for the slightly lower interface strength of the 3D-printed joint compared to that of the electron beam weld joint.

## 4. Conclusions

Based on a comparison of the surface morphology, microstructure, and mechanical properties of titanium alloy joints obtained through electron beam welding and laser additive manufacturing, the following conclusions have been drawn:

(1) A comparison of the surface morphology between the electron beam weld joint and the additively manufactured joint revealed that the surface of the additive manufacturing joint was smoother and flatter with less spatter.
(2) In the electron beam weld joint, microhardness increases progressively from the forging to the weld, accompanied by a gradual transformation in the microstructure. In contrast, the additively manufactured joint exhibits an abrupt increase in hardness from the forging to the additive section, with the microstructure undergoing a sudden change and the maximum microhardness reaching 355 HV.
(3) The maximum strength of the electron beam weld joint is observed at the weld top, with a peak tensile strength of 1211 MPa. The additively manufactured joint achieves a peak tensile strength of 1123 MPa. Both joints exhibit a combination of brittle and ductile fracture modes.
(4) The electron beam weld joint generates a pinning effect at the interface, which enhances the joint’s overall strength. In contrast, the additive manufacturing joint exhibits an adhesive effect near the interface, leading to reduced joint strength.

## Figures and Tables

**Table 1:** Welding parameters used for EBW of thick titanium plates and deposition parameters used for additive manufacturing.

Connection Methods	Machine Setting	Parameters
Electron beam welding	Accelerating voltage (kV)	150
	Focus current (mA)	2100~2200
	Beam current (mA)	120~150
	Welding speed (mm/s)	6~8
	Scanning pattern	Circle
	Scanning frequency (Hz)	500
Laser deposition	Laser cladding power (W)	3600~3900
	Scanning speed (mm/min)	900~1100
	Spot diameter (mm)	6~6.5
	Powder feed rate (g/min)	25~30

**Table 2:** Mechanical performance data for different regions of the two joints obtained in the experiments.

Zone	Tensile Strength (MPa)	Elongation (%)
Forged TC4	1314	23.805
Additive-manufactured TC4	1242	22.5
Top	1211	7.8
Middle	1165	7.3
Bottom	1183	7.6
Additive manufacturing joint	1123	5.9

## Data Availability

The original contributions presented in the study are included in the article, further inquiries can be directed to the corresponding author.
